# Prevalence of and Risk Factors for Hyperuricemia in Urban Chinese Check-Up Population

**DOI:** 10.1155/2024/8815603

**Published:** 2024-10-30

**Authors:** Tianxing Feng, Chaochen Li, Jiali Zheng, Yaqing Xu, Xiaoxiao Wang, Yisen Li, Yilei Wang, Beili Zhu, Li Zhao, Jiawei Yu

**Affiliations:** ^1^Department of Medical Affairs, Shanghai Clinical Research and Trial Center, Shanghai 201203, China; ^2^Department of Spine Surgery, The First People's Hospital of Nantong, Nantong 226001, Jiangsu, China; ^3^Department of Epidemiology and Biostatistics, School of Public Health, Shanghai Jiao Tong University School of Medicine, Shanghai 200025, China; ^4^Center for RNA Therapeutics, Houston Methodist Research Institute, Houston 77030, Texas, USA; ^5^Business Division of Health Check-Up, Rici Health Care Holdings Limited, Shanghai 200126, China; ^6^Department of Nephrology, Zhongshan Hospital of Fudan University, Shanghai 200032, China

**Keywords:** adult, Chinese, health check-up, hyperuricemia, prevalence, risk factor

## Abstract

**Background:** The prevalence of hyperuricemia is increasing globally. The health check-up population is a group of people dedicated to disease prevention in public health. This study aims to estimate the current prevalence of hyperuricemia among the health check-up population in economically developed areas of China using a healthcare database.

**Method:** Healthcare data from 48,988 subjects in 12 provinces of China who had an annual health check-up in 2021 were used. Hyperuricemia was defined as a serum urate level > 420 mmol/L and/or a history of physician-diagnosed gout. An alternative definition of serum urate level > 420 mmol/L in men and > 360 mmol/L in women was used. The stratified prevalence of hyperuricemia by sex, age, region, and comorbidity group was reported. The association between hyperuricemia and sex, age, region, and comorbidities was analyzed in the multivariate logistic regression model.

**Results:** In 2021, the sex- and age-adjusted prevalence of hyperuricemia was 13.6% in the total population (24.3% in men and 2.6% in women) based on the definition of serum urate level > 420 mmol/L. Regional prevalence varied considerably across the country, with the highest prevalence found in Fujian and the lowest in Liaoning Province (21.6% vs. 7.3%). Male sex, aging, hypertension, obesity, abdominal obesity, hypertriglyceridemia, and hypercholesterolemia were likely to be associated with hyperuricemia.

**Conclusions:** This is the largest study using a healthcare database to indicate the prevalence of hyperuricemia in a health check-up population in an economically developed area of China. The current prevalence among the Chinese health check-up population was substantial, with a higher prevalence in males and in the eastern region. Hyperuricemia and its comorbidities warrant greater attention in the developed areas of China.

## 1. Key Messages

This study investigated the up-to-date prevalence of hyperuricemia in 12 most developed provinces of China, using a health check-up population database including more than 3 million people in 2021.

It reported the prevalence using the definition of serum urate level > 420 mmol/L in both men and women and alternative definition of serum urate level > 420 mmol/L in men and > 360 mmol/L in women.

It also addressed likely risk factors of males, aging, hypertension, obesity, abdominal obesity, hypertriglyceridemia, and hypercholesterolemia associated with achieving hyperuricemia.

## 2. Background

Hyperuricemia is a pathological condition that is a direct cause of gout and uric acid nephropathy. Furthermore, hyperuricemia is also an independent risk factor for the development of obesity, chronic kidney disease (CKD), hypertension, type 2 diabetes, dyslipidemia, coronary heart disease, and stroke [1]. The prevalence of hyperuricemia among the Chinese population reported in many studies differs greatly due to selection bias of age, ethnicity, region, and comorbidities [2]. Three meta-analyses summarized the studies published after 2000 to obtain pooled prevalence of hyperuricemia in China of 13.3%, 16.4%, and 17.4%, respectively [3–5]. However, the included literature had a long-time span and could not reflect the current prevalence rate. Some studies used different diagnostic criteria for hyperuricemia, rendering the prevalence rates incomparable. Few nationwide surveys of the prevalence of hyperuricemia have been conducted. In addition, the prevalence of hyperuricemia is rising worldwide, including in China [5, 6]. However, it remains unknown how fast this trend has increased and what factors are associated with this high prevalence in China. To address this issue, we performed this large cross-sectional study to examine the latest prevalence using a health check-up database of urban Chinese adults in 12 provinces, among which 11 had a gross domestic product of more than 400 million RMB in 2021. We also examined the association of sex, age, and comorbidities of hypertension, dyslipidemia, obesity, diabetes, and CKD with the prevalence of hyperuricemia to address the likely associations with hyperuricemia.

## 3. Methods

### 3.1. Study Population

This was a cross-sectional study using a Chinese healthcare database. The database is managed by Rici Health Care Holdings Limited, China. Rici ranks in the top three private-owned national-chained health check-up agencies in China. The database was built in 2007 and included more than 3 million people in 2021. It provided a profile of the population aged 18–95 years old. The population covered by health check-ups comprised prehired, active, and retired employees from business clients, as well as individual walk-in visitors. Since China does not have a sophisticated family physician system, the early screening, preliminary diagnosis, and referral of patients are achieved mainly through annual health check-ups. A study showed that 60.8% of adults in Beijing received at least one health check-up every 2 years [7]. The health check-up population mainly represented a group of employed and retired people who cared about their own health status. The health check-up data included subjects' demographic information, past medical history, physical measurements, clinical laboratory test results, and medical imaging results. This database had a larger population aged 30–59 years old and a less population aged over 60 years old than the general population. This enabled us to estimate the prevalence of hyperuricemia among a health check-up population in an economically developed area of China.

The study subjects came from the health check-up branches allocated to the capital cities of 12 provinces, including Liaoning, Beijing, Shandong, Jiangsu, Shanghai, Zhejiang, Fujian, and Guangdong in the eastern region, Anhui, Hubei, and Hunan in the middle region, and Sichuan in the western region. We analyze a subset of the original data to balance the proportional differences between regions in our study. To produce representative national results, we adopted a sampling strategy, which randomly selected observations from the original database based on the population proportions of all regions at the end of 2021 published by the National Bureau of Statistics [8]. The sample size for Shandong was set as the base number, and the sample sizes for other provinces were calculated according to the corresponding proportion. The subjects were then included after random sampling from the database. Random numbers were generated for each individual in different provinces, and subjects were then included in the study after sorting by random numbers from small to large. Approval for this research by the ethical review committee of Rici Healthcare approved this study. Informed consent of each subject was not required because this study used only unlinkable anonymized data.

### 3.2. Definition of Hyperuricemia

This study reported the prevalence of hyperuricemia by using two different criteria. A sex-specific definition of hyperuricemia was a one-time fasting serum urate level > 420 mmol/L (7.0 mg/dL) in men and > 360 mmol/L (6.0 mg/dL) in women and/or a gout diagnosis according to past medical history obtained onsite [9]. The sex-identical definition of hyperuricemia was a one-time fasting serum urate level > 420 mmol/L and/or a history of physician-diagnosed gout [10, 11]. The definition of hyperuricemia is nowadays widely recognized to be related to the crystallization threshold, namely, 6.0 mg/dL which is the same in males and females. We used two definitions because the sex-specific definition is more frequently used in the epidemiology statistics [2, 12]. In this study, the crude and stratified prevalence of hyperuricemia was reported by these two definitions. A loess curve of the hyperuricemia prevalence trend and risk factors and a multivariate logistic regression model of risk factors for hyperuricemia were performed by using the same definition for both sexes.

### 3.3. Demographic and Other Covariates

Demographic characteristics of participants included sex, age, and region. A standard protocol of anthropometric measurements, blood pressure measurement, and blood specimen collection and processing were followed. All the data were recorded in the electronic health record system. Past medical history was ascertained by a positive response to the following question by trained physicians: “Has a doctor ever told you that you have hypertension, diabetes, or chronic kidney disease?” Blood pressure was measured twice at 5-min intervals using an Omron HBP-9020 digital sphygmomanometer. The mean of the two readings was recorded. Hypertension was defined as a systolic blood pressure (SBP) ≥ 140 mmHg, a diastolic blood pressure (DBP) ≥ 90 mmHg, or a history of physician-diagnosed hypertension [13]. Height and weight were measured by a Woshen WS-H300 automatic digital physician scale. Waist and hip circumferences were measured manually based on a standard protocol. Body mass index (BMI) was calculated as weight in kilograms (kg) over height in centimeters (cm) squared and was categorized as normal (< 24.0 kg/m^2^), overweight (24.0–27.9 kg/m^2^), and obese (> 28.0 kg/m^2^) as per the guidelines of the Chinese population [14]. The waist–hip ratio (WHR) was calculated as waist circumference in centimeters (cm) over hip circumference in centimeters (cm). Abdominal obesity was defined as waist circumference ≥ 90 cm in men and ≥ 85 cm in women or a WHR of men and women ≥ 1.0 [14]. Fasting blood glucose (FBG), hemoglobin A1c (HbA1c), serum total cholesterol (TC), triglyceride (TG), low-density lipoprotein cholesterol (LDL-C), high-density lipoprotein cholesterol (HDL-C), serum creatinine (SCr), and serum urate were tested in a fasting state. The TC level was categorized as normal (<5.2 mmol/L), borderline elevated (5.2–6.2 mmol/L), and hypercholesterolemia (≥6.2 mmol/L). The TG level was categorized as normal (< 1.7 mmol/L), borderline elevated (1.7–2.3 mmol/L), and hypertriglyceridemia (≥ 2.3 mmol/L) [15]. Diabetes was defined as FBG ≥ 7.0 mmol/L or HbA1c ≥ 6.5% and/or a history of physician-diagnosed diabetes [16]. Measurements of SCr were used directly for the calculation of estimated glomerular filtration rate (eGFR) with the CKD-EPI quotation. CKD was defined as an eGFR of < 60 mL/min per 1.73 m^2^ and/or a history of physician-diagnosed CKD [17].

### 3.4. Statistical Analyses

The crude prevalence of hyperuricemia was reported in the total study population, and the stratified prevalence was reported by sex, age, region, and comorbidities. To examine the shape of the relationship between the prevalence and covariates, including age, SBP, DBP, eGFR, FBG, HbA1c, TC, TG, LDL-C, HDL-C, BMI, and WHR, we drew a lowess curve to describe the trend [18]. Logistic regression was used to screen the factors purported to be associated with hyperuricemia, adjusting for sex, age, and comorbidities. The results were reported as odds ratios (ORs) with 95% confidence intervals. Covariates included in the multivariate logistic regression model were sex, age, region, hypertension, diabetes, TG level, TC level, obesity, abdominal obesity, and eGFR result. The reported age- and sex-adjusted prevalence was assessed using a direct-adjustment formula that weights the age- and sex-specific rates observed in a population of interest by the proportion of each age group in a standard population. Stata 16 (StataCorp, College Station, TX) was used for all analyses. Tableau Desktop (Tableau Software, Seattle, WA) was used for the data visualization.

## 4. Results

### 4.1. Prevalence of Hyperuricemia

A total of 48,988 subjects with complete medical records were included. Using the sex-specific definition, the crude prevalence of hyperuricemia was 13.3% overall (25.2% among men and 2.1% among women). Using the age and sex distribution of the nation in 2021 (China Statistical Yearbook 2021), the age-adjusted prevalence of hyperuricemia among the total population, men, and women was 13.6%, 24.3%, and 2.6%, respectively. The prevalence of hyperuricemia decreased with age in men, with the highest prevalence observed in the 18–29-year-old age group. The age trend was opposite in women, with a higher prevalence among those aged 50 years or older (Table 1). The older individuals have lower odds of developing the hyperuricemia, which is a contradictory finding to current literature in China [5]. It is possible that older patients receiving allopurinol are included as individuals with normal uric acid. The crude prevalence of hyperuricemia in the eastern region (13.8%) was slightly higher than that in the western region (11.2%). Among the 12 provinces in this study, Fujian Province had the highest prevalence (21.6%), and Liaoning Province had the lowest prevalence (7.3%) (Table 2).

### 4.2. Risk Factors Associated With the Prevalence of Hyperuricemia

**Figure 1:**
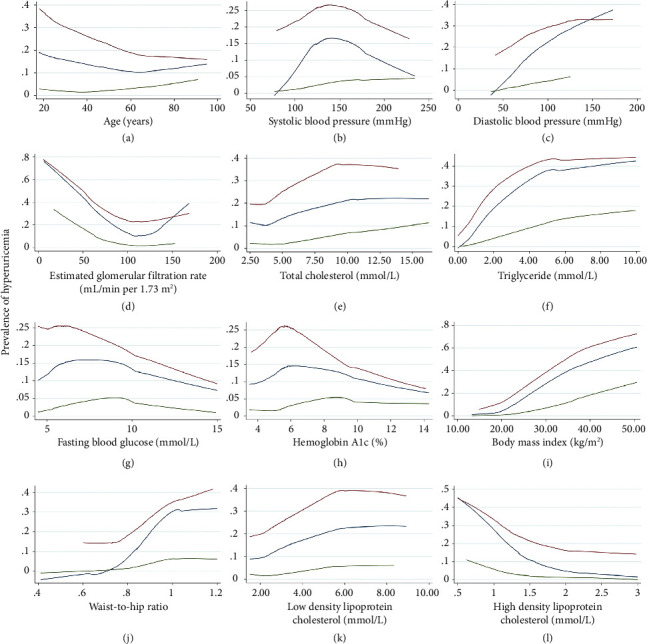
Lowess curve of the hyperuricemia prevalence trend and risk factors by sex. Hyperuricemia was defined as a serum urate level of > 420 mmol/L in both men and women in this figure. The description of the unadjusted association of the hyperuricemia prevalence trend and risk factors was composed of two paired graphs. The blue line represents total participants, the red line represents men, and the green line represents women.

The prevalence of hyperuricemia increased as age, DBP, TC level, TG level, LDL-C level, BMI, and WHR increased, and the prevalence decreased as the HDL-C level increased (Figure 1). The trend in the prevalence of SBP, FBG, HbA1c, TC, and eGFR reached a turning point. The prevalence of eGFR exhibited an inflection point at approximately 120 mL/min per 1.73 m^2^. When eGFR < 120 mL/min per 1.73 m^2^, the prevalence decreased with increasing eGFR (Figure 1(d)). When FBG ≥ 7.0 mmol/L or HbA1c ≥ 6.5%, the prevalence of hyperuricemia decreased with increasing risk factor value, and when FBG < 7.0 mmol/L or HbA1c < 6.5%, the prevalence changed in the opposite direction (Figures 1(g) and 1(h)).

After adjustment for age, region, hypertension, diabetes, TG level, TC level, obesity, abdominal obesity, and eGFR result, women were found to have 10-fold lower odds of having hyperuricemia than men according to sex-identical definition (adjusted OR 0.10 [95% CI 0.09–0.11]). Accordingly, the odds of having hyperuricemia decreased with aging. Subjects who lived in the middle region had a lower odds of hyperuricemia than those in the eastern region (adjusted OR 0.64 [95% CI 0.59–0.70]). Compared with the healthy subjects, the subjects with hypertension, hypertriglyceridemia, hypercholesterolemia, obesity, abdominal obesity, and stage 4 CKD had higher odds of exhibiting hyperuricemia. The adjusted OR and 95% CI were 1.15 (1.05–1.27), 2.48 (2.29–2.69), 1.22 (1.10–1.35), 2.19 (1.95–2.46), 1.33 (1.22–1.45), and 12.55 (4.74–33.24), respectively. In contrast, diabetes patients had lower odds of having hyperuricemia (adjusted OR 0.74 [95% CI 0.65–0.84]) (Table 3).

## 5. Discussion

Using a healthcare database of an urban Chinese health check-up population in 12 provinces, we found that the sex- and age-adjusted prevalence of hyperuricemia in 2021 was 13.6% overall using a definition of serum urate level > 420 mmol/L in both men and women and/or a history of physician-diagnosed gout. However, the overall prevalence would be raised to 16.0% based on a sex-specific definition, which was more comparable to the result in a similar study published in 2015. It included an 18–59-year-old population from 15 provinces in China and reported that the adjusted prevalence of hyperuricemia was 9.8% overall (15.1% among men and 5.8% among women) [19]. This may reveal the increasing trend of hyperuricemia in China. Logistic regression analysis showed that male sex, aging, hypertension, hypertriglyceridemia, hypercholesterolemia, obesity, abdominal obesity, and advanced CKD stage were likely risk factors associated with achieving hyperuricemia. Diabetes and eGFR > 120 mL/min per 1.73 m^2^ were shown to be protective against hyperuricemia.

The prevalence of hyperuricemia is increasing globally but is stable in some developed countries [6]. A meta-analysis reported that the estimated prevalence of hyperuricemia increased from 8.5% to 18.4% from 2001 to 2017 in China [5]. The United States reported a prevalence of hyperuricemia of 20.1% in 2015–2016, which was steady at least from 2007–2008 [9]. Similarly, a prevalence of 14.2% in 2014 was reported in Japan but remained stable from 2010–2014 [20]. Other Asian regions reported a prevalence of 11.4% in Korea in 2018 [21]. Our study reported that the prevalence of hyperuricemia in China was close to the prevalence in the Asian population, but the previous increasing trend was faster than that in other countries. This result may be related to economic development, metabolic disease increases, and lifestyle changes, such as increasing consumption of beer, smoking, and red meat [6]. It is worth noting that the study in the United States and Korea used a sex-specific definition of hyperuricemia. Direct comparisons between studies can only be made with caution due to differing case definitions and many other factors, such as different populations.

### 5.1. Prevalence by Demographic Factors

The prevalence of hyperuricemia was influenced by sex, age, ethnicity, and region. Using a sex-identical definition of hyperuricemia, our study reported a sex- and age-adjusted prevalence of 24.3% among men and 2.6% among women. Other Asian countries reported a prevalence of 26.8% and 0.9% among Japanese men and women [20] and 17.0% and 5.9% among Korean men and women, respectively (using serum urate levels > 357 mmol/L in women) [21]. The US reported a prevalence of 5.1% among women, which was much higher than that in the Asian population [9]. Regarding the age trend, a U-shaped association was observed between hyperuricemia and age overall. The shape was formed by a decreasing prevalence trend with aging in men and a converse change in women. This was due to the uricosuric effects of estrogen after menopause in women, causing a tripled prevalence in women older than 50 years. The prevalence of hyperuricemia also varied among ethnicities, although our study did not analyze this covariant. The prevalence of hyperuricemia was reported to be 21.5% in Tibetan [22], 8.38% in Uighur [23], and 11.3% in Mongolian [24] in China. However, the prevalence of hyperuricemia among different ethnic groups in China cannot be compared due to differences in research methods, age distribution, and comorbidities among the reports. Additionally, this study confirmed that the prevalence varied considerably across the country, with a higher prevalence in the eastern region than in the western region (adjusted OR 0.87 [95% CI 0.76–1.00]). In comparison to a previous study that found a prevalence of 13.7% in Northeastern Chinese provinces, our study, which encompassed provinces mostly in China's eastern and southern regions, found a higher prevalence of hyperuricemia [25]. This result was consistent with the fact that economically developed regions and urban areas had a higher prevalence than economically developing regions and rural areas [19]. However, we observed that the prevalence of hyperuricemia did not have a linear relationship with the level of economic development. The prevalence in Fujian Province was 3 times that in Liaoning Province, even though both provinces were eastern provinces. We speculated that the difference may be attributed to living habits, especially dietary habits.

### 5.2. Risk Factors and Comorbidities

Abundant studies have confirmed the association between hyperuricemia and comorbidities, including hypertension, dyslipidemia, obesity, and CKD [6]. Among these comorbidities, our study showed that CKD was associated with the highest risk of hyperuricemia, with an adjusted OR of 8.43 (95% CI 6.62–10.74) in stage 3 CKD and 12.55 (95% CI 4.74–33.24) in stage 4 CKD. This result was consistent with previous reports because a decrease in uric acid clearance that accompanies decreased renal function increased blood urate levels [26]. We found that the prevalence increased with increasing eGFR when eGFR > 120 mL/min per 1.73 m^2^. Those whose eGFR was > 120 mL/min per 1.73 m^2^ were mainly younger than 40 years, so the higher prevalence of hyperuricemia in the younger adults demonstrated the opposite trend. Of the variety of dyslipidemias, hypertriglyceridemia (adjusted OR 2.48 [95% CI 2.29–2.69]) was associated with a higher risk of hyperuricemia than hypercholesterolemia (adjusted OR 1.22 [95% CI 1.10–1.35]). Hypertriglyceridemia is the most common risk factor for hyperuricemia, but different correlations were reported for other parameters in the lipid profile. A study of US adults reported that higher TC and LDL-C and decreased HDL-C were associated with elevated serum urate levels [27]. Similarly, a study of a Chinese population in Xinjiang revealed that higher TC was associated with hyperuricemia, but the association between higher LDL-C or lower HDL-C and hyperuricemia was not significant [28]. As a result of dyslipidemia, an elevated visceral adiposity index was found to increase the risk of hyperuricemia in middle-aged and elderly populations in the Chinese [29]. More proof is needed to clarify the relationship between serum lipid profiles and hyperuricemia. Obesity was found to be a cause of hyperuricemia [1]. We found that both BMI and WHR had a good linear relationship with the prevalence of hyperuricemia. The odds of hyperuricemia in people with a BMI > 28.0 kg/m^2^ (adjusted OR 2.19 [95% CI 1.95–2.46]) were higher than those of people with abdominal obesity (adjusted OR 1.33 [95% CI 1.22–1.45]). In addition, the prevalence of hyperuricemia was increased in IFG patients but decreased in diabetes patients in our study. This result was explained by the fact that glycosuria increased urine excretion of urate [30].

### 5.3. Strength and Limitation

This study was based on a large healthcare database covering 12 provinces to estimate the up-to-date prevalence of hyperuricemia in the economically developed area of China. The health check-up population is a group of people dedicated to disease prevention in public health. The omission of unemployed individuals, housewives, and nonutilizers of health check-ups limits that the prevalence of hyperuricemia in the health check-up population cannot be equal to that of the general population. The study used a sex-identical definition as well as a sex-specific definition of hyperuricemia to report the prevalence, facilitating comparison with other prevalence studies. This study has several limitations. First, 65.4% of the study population inhabited the eastern region of China, and only 10.8% inhabited the western region, which was less developed than the eastern part. In addition, all the health check-up clinics were located in the capital cities of the included provinces. Thus, the rural population was not included. The ethnicity of the subjects was not recorded, but all 12 provinces included were mainly inhabited by individuals of the Han nationality. All these factors mentioned above would lead to an overestimation of the prevalence of hyperuricemia when the data were generalized as the prevalence of hyperuricemia among the entire Chinese population. In contrast, the definition of hyperuricemia was defined by a one-time blood test and a history of gout. The past medical history of gout was based on a physician-diagnosed diagnosis, which was prone to recall bias. The use of allopurinol, febuxostat, and several kinds of diuretics as well as SGLT2 inhibitors will mask the presence of hyperuricemia. These factors may have caused an underestimation of the prevalence of hyperuricemia. Besides, information on lifestyle, such as red meat intake, alcohol consumption, and smoking, was not available in the health check-up database. These factors have been confirmed to have a positive influence on the prevalence of hyperuricemia in the literature [31]. For instance, residents in urban Fujian and Guangdong provinces had a higher consumption of seafood, which may have contributed to the high prevalence in these two provinces [32]. However, such information is not sufficient to explain the lower prevalence of hyperuricemia in Zhejiang Province, despite the higher consumption of seafood than that in Fujian Province. This difference in prevalence is also seen with other related psychosocial and nutritional factors that are not assessed at health check-ups. Finally, this cross-sectional study cannot reflect how fast the prevalence of hyperuricemia has changed in China. Further research on the prevalence trend would be helpful to address this issue.

## 6. Conclusions

This is the largest study using a healthcare database to assess the prevalence of hyperuricemia among a health check-up population in economically developed areas of China. The current prevalence among the Chinese health check-up population was substantial, with a higher prevalence among males and in the eastern region. The definition of hyperuricemia needs to be harmonized for easy data comparison between different studies. Male sex, aging, hypertension, obesity, abdominal obesity, hypertriglyceridemia, and hypercholesterolemia were likely risk factors for hyperuricemia. Considering the recommendation of urate-lowering therapy in asymptomatic hyperuricemia as per the Chinese guidelines [2], it is important to be aware of the up-to-date prevalence of hyperuricemia among the Chinese population.

## Figures and Tables

**Table 1:** Demographic characteristics and crude prevalence of hyperuricemia using different definitions in the analysis samples.

	Participants included in this study, *n* (%)			Participants with hyperuricemia using a sex-specific definition, *n* (%)^1^			Participants with hyperuricemia using a sex-identical definition, *n* (%)^1^		
	Total	Men	Women	Total	Men	Women	Total	Men	Women
Total population	48,988 (100)	23,742 (48.5)	25,246 (51.5)	7820 (16.0)	5980 (25.2)	1840 (7.3)	6519 (13.3)	5980 (25.2)	539 (2.1)
Age, years									
18–29	8237 (16.8)	3895 (8.0)	4342 (8.9)	1628 (19.8)	1258 (32.3)	370 (8.5)	1346 (16.3)	1258 (32.3)	88 (2.0)
30–39	14,226 (29.0)	7159 (14.6)	7067 (14.4)	2447 (17.2)	2063 (28.8)	384 (5.4)	2182 (15.3)	2063 (28.8)	119 (1.7)
40–49	9671 (19.7)	4740 (9.7)	4931 (10.1)	1388 (14.4)	1151 (24.3)	237 (4.8)	1209 (12.5)	1151 (24.3)	58 (1.2)
50–59	10,786 (22.0)	4931 (10.1)	5855 (12.0)	1465 (13.6)	977 (19.8)	488 (8.3)	1131 (10.5)	977 (19.8)	154 (2.6)
60–69	4834 (9.9)	2348 (4.8)	2486 (5.1)	695 (14.4)	409 (17.4)	286 (11.5)	505 (10.4)	409 (17.4)	96 (3.9)
70–79	1071 (2.2)	566 (1.2)	505 (1.0)	167 (15.6)	105 (18.6)	62 (12.3)	122 (11.4)	105 (18.6)	17 (3.4)
≥ 80	163 (0.3)	103 (0.2)	60 (0.1)	30 (18.4)	17 (16.5)	13 (21.7)	24 (14.7)	17 (16.5)	7 (11.7)
Region2									
Eastern region	32,021 (65.4)	15,476 (31.6)	16,545 (33.8)	5341 (16.7)	4018 (26.0)	1323 (8.0)	4426 (13.8)	4018 (26.0)	408 (2.5)
Middle region	11,686 (23.9)	5879 (12.0)	5807 (11.9)	1765 (15.1)	1409 (24.0)	356 (6.1)	1504 (12.9)	1409 (24.0)	95 (1.6)
Western region	5281 (10.8)	2387 (4.9)	2894 (5.9)	714 (13.5)	553 (23.2)	161 (5.6)	589 (11.2)	553 (23.2)	36 (1.2)
Comorbidities									
Hypertension	8369 (17.1)	4848 (9.9)	3521 (7.2)	1716 (20.5)	1296 (26.7)	420 (11.9)	1429 (17.1)	1296 (26.7)	133 (3.8)
Hypertriglyceridemia	7376 (15.1)	5413 (11.0)	1963 (4.0)	2106 (38.9)	372 (19.0)	372 (19.0)	2245 (30.4)	2106 (38.9)	139 (7.1)
Obesity	6343 (12.9)	4345 (8.9)	1998 (4.1)	1709 (39.3)	395 (19.8)	395 (19.8)	1855 (29.2)	1709 (39.3)	146 (7.3)
Hypercholesterolemia	5454 (11.1)	2630 (5.4)	2824 (5.8)	825 (31.4)	275 (9.7)	275 (9.7)	924 (16.9)	825 (31.4)	99 (3.5)
Diabetes	3085 (6.3)	1956 (4.0)	1129 (2.3)	403 (20.6)	156 (13.8)	156 (13.8)	460 (14.9)	403 (20.6)	57 (5.0)
Chronic kidney disease	463 (0.9)	379 (0.8)	1129 (0.2)	194 (51.2)	35 (41.7)	35 (41.7)	214 (46.2)	194 (51.2)	20 (23.8)

^1^The sex-specific definition of hyperuricemia was a serum urate level of > 420 mmol/L in men and > 360 mmol/L in women. The sex-identical definition of hyperuricemia was a serum urate level of > 420 mmol/L in both men and women.

^2^Eastern region includes Liaoning, Beijing, Shandong, Jiangsu, Shanghai, Zhejiang, Fujian, and Guangdong provinces. The middle region includes Anhui, Hunan, and Hubei Provinces. The western region includes Sichuan Province.

**Table 2:** Crude province prevalence of hyperuricemia using different definitions in the analysis sample.

Province	Percentage of the Chinese population, %	Number of participants, *n* (%)	Participants with hyperuricemia using a sex-specific definition, *n* (%)^1^			Participants with hyperuricemia using a sex-identical definition, *n* (%)^1^		
			All	Men	Women	All	Men	Women
Total	55.0	48,988 (100)	7820 (16)	5980 (25.2)	1840 (7.3)	6519 (13.3)	5980 (25.2)	539 (2.1)
Guangdong	8.9	7952 (16.2)	1719 (21.6)	1259 (31.7)	460 (11.6)	1400 (17.6)	1257 (31.7)	143 (3.6)
Shandong	7.2	6402 (13.1)	1094 (17.1)	816 (25.7)	278 (8.6)	938 (14.7)	816 (25.7)	122 (3.8)
Jiangsu	6.0	5342 (10.9)	661 (12.4)	543 (20.4)	118 (4.4)	566 (10.6)	531 (20.0)	35 (1.3)
Sichuan	5.9	5281 (10.8)	714 (13.5)	553 (23.2)	161 (5.6)	589 (11.2)	553 (23.2)	36 (1.2)
Hunan	4.7	4194 (8.6)	391 (9.3)	306 (15.1)	85 (3.9)	326 (7.8)	306 (15.1)	20 (0.9)
Zhejiang	4.6	4069 (8.3)	449 (11)	374 (17.9)	75 (3.8)	386 (9.5)	374 (17.9)	12 (0.6)
Anhui	4.3	3848 (7.9)	645 (16.8)	507 (26.6)	138 (7.1)	547 (14.2)	507 (26.6)	40 (2.1)
Hubei	4.1	3644 (7.4)	729 (20)	596 (30.6)	133 (7.8)	631 (17.3)	596 (30.6)	35 (2.1)
Liaoning	3.0	2688 (5.5)	262 (9.7)	170 (17.7)	92 (5.3)	195 (7.3)	170 (17.7)	25 (1.4)
Fujian	2.9	2619 (5.4)	715 (27.3)	522 (41)	193 (14.3)	565 (21.6)	522 (41.0)	43 (3.2)
Shanghai	1.8	1568 (3.2)	279 (17.8)	208 (28.9)	71 (8.4)	227 (14.5)	208 (28.9)	19 (2.2)
Beijing	1.6	1381 (2.8)	162 (11.7)	126 (20.1)	36 (4.8)	135 (9.8)	126 (20.1)	9 (1.2)

^1^The sex-specific definition of hyperuricemia was a serum urate level of > 420 mmol/L in men and > 360 mmol/L in women. The sex-identical definition of hyperuricemia was a serum urate level of > 420 mmol/L in both men and women.

**Table 3:** Multivariable models of risk factors for hyperuricemia using sex-identical definition among 48,988 participants in China, 2021.

	Participants with hyperuricemia, *n* (%)	Unadjusted odds ratio	Fully adjusted odds ratio (95% CI)^1^
Sex			
Men	5980 (25.2)	0.33	Reference
Women	539 (2.1)	0.02	0.10 (0.09–0.11)
Age, years			
18–29	1346 (16.3)	0.20	Reference
30–39	2182 (15.3)	0.18	0.62 (0.57–0.69)
40–49	1209 (12.5)	0.14	0.40 (0.36–0.45)
50–59	1131 (10.5)	0.12	0.31 (0.28–0.35)
60–69	505 (10.4)	0.12	0.27 (0.24–0.32)
70–79	122 (11.4)	0.13	0.22 (0.17–0.28)
≥ 80	24 (14.7)	0.17	0.18 (0.11–0.31)
Region			
Eastern region	4426 (13.8)	0.16	Reference
Middle region	1504 (12.9)	0.15	0.64 (0.59–0.70)
Western region	589 (11.2)	0.13	0.87 (0.76–1.00)
Comorbidities			
Hypertension			
No	2266 (9.4)	0.10	Reference
Prehypertension	2824 (17)	0.21	1.14 (1.06–1.23)
Yes	1429 (17.1)	0.21	1.15 (1.04–1.26)
Diabetes			
No	5436 (12.8)	0.15	Reference
IFG	623 (18.6)	0.23	1.13 (1.00–1.27)
Yes	460 (14.9)	0.18	0.74 (0.65–0.84)
Triglyceride level, mmol/L			
Normal	2922 (8.4)	0.09	Reference
1.7–2.3	1352 (20.1)	0.25	1.76 (1.62–1.93)
≥ 2.3	2245 (30.4)	0.44	2.48 (2.29–2.69)
Total cholesterol level, mmol/L			
< 5.2	3536 (11.9)	0.13	Reference
5.2–6.2	2059 (15.0)	0.18	1.05 (0.98–1.13)
≥ 6.2	924 (16.9)	0.20	1.22 (1.10–1.35)
Obesity			
No	1626 (6.4)	0.07	Reference
Overweight	3038 (17.8)	0.22	1.62 (1.49–1.75)
Obese	1855 (29.2)	0.41	2.19 (1.95–2.46)
Abdominal obesity2			
No	3315 (10.5)	0.12	Reference
Yes	2292 (26.8)	0.37	1.33 (1.22–1.45)
eGFR, ml/min per 1.73 m^2^			
≥ 90	4516 (11.3)	0.13	Reference
60–89	1789 (21.3)	0.27	2.08 (1.91–2.25)
30–59	204 (46.2)	0.86	8.43 (6.62–10.74)
15–29	10 (47.6)	0.91	12.55 (4.74–33.24)

Abbreviations: 95% CI, 95% confidence interval; eGFR, estimated glomerular filtration rate; IFG, impaired fasting glucose.

^1^Fully adjusted odds ratio was adjusted for sex, age, region, and all the other comorbidities. The widths of the confidence intervals were adjusted for multiple comparisons. Hyperuricemia was defined as a serum urate level of > 420 mmol/L in both men and women.

^2^Abdominal obesity was analyzed in 40,074 participants.

## Data Availability

Raw data were generated at Rici Healthcare Holdings Limited, China. Derived data supporting the findings of this study are available from the corresponding authors upon reasonable request.
